# Changes in repetitive negative thinking and stress perception mediate treatment effects of a transdiagnostic exercise intervention

**DOI:** 10.1017/S0033291725103085

**Published:** 2026-01-09

**Authors:** Anna Katharina Frei, Thomas Studnitz, Britta Seiffer, Jana Welkerling, Johanna-Marie Zeibig, Eva Herzog, Mia Maria Günak, Thomas Ehring, Keisuke Takano, Tristan Nakagawa, Leonie Sundmacher, Sebastian Himmler, Stefan Peters, Anna Lena Flagmeier, Lena Zwanzleitner, Ander Ramos-Murguialday, Gorden Sudeck, Sebastian Wolf

**Affiliations:** 1 University of Tübingen: Eberhard Karls Universitat Tubingen, Germany; 2 University of Munich: Ludwig-Maximilians-Universitat Munchen, Germany; 3 German Center for Mental Health (DZPG), Munich Site; 4 National Institute of Advanced Industrial Science and Technology Tsukuba Center, Japan; 5 Munich Center for Health Economics and Policy; 6 Technical University Munich: Technische Universitat Munchen, Germany; 7 German Association for Health-related Fitness and Exercise Therapy (German: DVGS), Hurth-Efferen, Germany; 8 Bundeswehr University Munich: Universitat der Bundeswehr Munchen, Germany; 9 AOK Baden-Wurttemberg, Germany; 10 Techniker Krankenkasse, Germany; 11 Tecnalia, Basque Research and Technology Alliance, San Sebastian, Spain; 12 Athenea Neuroclinics, San Sebastian, Spain

**Keywords:** exercise intervention, mental disorders, transdiagnostic, underlying mechanisms

## Abstract

**Background:**

Exercise improves stress perception and sleep quality and reduces repetitive negative thinking in patients with various mental disorders. However, it is unclear whether changes in these processes mediate treatment effects on psychopathology in a transdiagnostic sample.

**Methods:**

Physically inactive adult outpatients with depressive disorders, agoraphobia, panic disorder, post-traumatic stress disorder, and/or nonorganic primary insomnia were randomly allocated to ImPuls – a 6-month transdiagnostic group exercise intervention – plus treatment-as-usual (*n* = 198), or to a treatment-as-usual alone control group (*n* = 201) at 10 study sites between March 2021 and May 2022. The primary outcome was global symptom severity; perceived stress, repetitive negative thinking, and sleep quality were included as mediators. All variables were assessed at baseline, 6 months, and 12 months using validated rating scales. As a secondary analysis of an RCT, intention-to-treat analyses were performed using structural equation modeling to test whether changes in stress perception, repetitive negative thinking, and sleep quality mediate treatment effects on changes in global symptom severity in two path models (from baseline to 6 and 12 months, respectively).

**Results:**

Treatment effects on global symptom severity were fully mediated by changes in perceived stress (6 months: β = −0.99, *p* = .024; 12 months: β = −1.28, *p* = .014) and repetitive negative thinking (6 months: β = −1.34, *p* = .004; 12 months: β = −0.94, *p* = .024).

**Conclusions:**

Our results suggest that changes in perceived stress and repetitive negative thinking may be key transdiagnostic mechanisms underlying the treatment effect of exercise on global symptom severity.

## Introduction

The disorder-specific effects of exercise on mental health conditions such as depression, agoraphobia, panic disorder, post-traumatic stress disorder (PTSD), and nonorganic primary insomnia are well established (Banno et al., 2018; Noetel et al., 2024; Ramos-Sanchez et al., 2021; Rosenbaum et al., 2015). Recent findings from a large-scale randomized controlled trial further indicate that ImPuls – a 6-month transdiagnostic group exercise intervention – also reduces global transdiagnostic symptom severity in an outpatient sample diagnosed with these mental disorders (Wolf et al., 2024). However, the mechanisms underlying this transdiagnostic effect remain unclear. The current study represents a secondary analysis of the ImPuls trial, focusing on three key processes common to these conditions: stress perception, repetitive negative thinking, and sleep quality.

Stress, defined as a discrepancy between environmental or internal demands and an individual’s adaptive resources (Monat & Lazarus, 1991) has been found to be involved in the development and maintenance of depression, agoraphobia, panic disorder, PTSD, and insomnia (Healey et al., 1981; Kendler, Karkowski, & Prescott, 1999; Moreno-Peral et al., 2014; Schock et al., 2016). Specifically, subjective stress perception refers to an individual’s cognitive appraisal of stressors and their perceived ability to cope with them (Lazarus & Folkman, 1984). Repetitive negative thinking is a common dysfunctional style of responding to stress or adversity that is characterized as being repetitive, partly intrusive, difficult to disengage from, unproductive and capturing mental capacity (Ehring et al., 2011). It is highly prevalent across various mental health disorders (Ehring & Watkins, 2008), predicts comorbidity (McEvoy, Watson, Watkins, & Nathan, 2013), and contributes transdiagnostically to the development and maintenance of psychopathology (Moulds, Bisby, Wild, & Bryant, 2020; Spinhoven, van Hemert, & Penninx, 2018). Similarly, reduced sleep quality is a common feature of the disorders of interest here that is also prospectively related to their development and maintenance (Freeman et al., 2020) and is associated with increased symptom severity (Belleville, Guay, & Marchand, 2009; Kallestad et al., 2012). In sum, stress perception, repetitive negative thinking, and reduced sleep quality represent key transdiagnostic processes (Dalgleish, Black, Johnston, & Bevan, 2020; Sauer-Zavala et al., 2017) involved in the onset and maintenance of depression, agoraphobia, panic disorder, PTSD, and nonorganic primary insomnia, and are related to symptom severity. Notably, these processes are interrelated and mutually influence each other in the perpetuation of mental disorders. For example, self-reported stressful life events are associated with subsequent increases in repetitive negative thinking, which is widely recognized as a maladaptive coping response to stress (Michl, McLaughlin, Shepherd, & Nolen-Hoeksema, 2013; Ward, Lyubomirsky, Sousa, & Nolen-Hoeksema, 2003). Some individuals engage in repetitive negative thinking at bedtime, which is associated with poor subjective sleep quality (Takano, Iijima, & Tanno, 2012) and shorter sleep duration (Nota & Coles, 2018). Compromised sleep can in turn undermine adaptive coping with stressors, reinforcing clinical symptoms in a self-perpetuating vicious cycle.

There is emerging evidence that exercise influences these transdiagnostic factors. Recent studies suggest that exercise can reduce stress perception, repetitive negative thinking, and enhance sleep quality in both healthy individuals (Klaperski, 2018; Wang & Boros, 2021) and disorder-specific samples (Lederman et al., 2019; Olson, Brush, Ehmann, & Alderman, 2017). However, evidence remains limited on whether these factors mediate the effects of exercise interventions on psychopathology in transdiagnostic samples. Notably, the use of a transdiagnostic sample is conceptually grounded in recent shifts toward a transdiagnostic understanding of psychopathology (Cuijpers et al., 2023; Dalgleish et al., 2020). Depression, agoraphobia, panic disorder, PTSD, and insomnia frequently co-occur and share underlying etiological and maintenance mechanisms, making disorder-specific research potentially inefficient and conceptually restrictive by overstating disorder-specific differences in treatment effects and mechanisms (Cuijpers et al., 2023). Importantly, a transdiagnostic approach allows for the examination of shared mechanisms – specifically perceived stress, repetitive negative thinking, and sleep quality – across diagnostic boundaries. In contrast, using disorder-specific measures would reduce sensitivity to detect changes across the entire sample and require multiple subgroup analyses, thereby diminishing statistical power. From a clinical perspective, transdiagnostic treatments offer greater efficiency by enabling the simultaneous treatment of diverse patient groups and by allowing for unified therapist training.

Understanding underlying transdiagnostic mechanisms provides a foundation for evidence-based frameworks that map the pathways linking interventions to clinical improvements. Identifying mediators allows interventions to be refined and optimized by targeting the most relevant mechanisms, thereby potentially enhancing treatment efficacy. Despite these advantages, underlying mechanisms remain poorly understood, even in disorder-specific samples. Indeed, a recent meta-review of disorder-specific studies found no meta-analytic evidence for psychosocial or behavioral mechanisms explaining the effects of exercise on mental health outcomes in individuals with mental disorders (Vancampfort et al., 2025). Although psychological constructs such as self-efficacy or self-esteem have been proposed as potential mediators, empirical findings – particularly from clinical samples – are still scarce (Bendau et al., 2024), and few studies have investigated underlying mechanisms in transdiagnostic samples. More broadly, whereas various hypotheses have been proposed to explain how and why exercise reduces transdiagnostic symptomatology of mental disorders (Jacquart et al., 2019), empirical support remains limited.

A recent preliminary study investigating the effects of exercise on stress in a transdiagnostic sample – including individuals with depressive and anxiety disorders, insomnia, and attention deficit hyperactivity disorder – found a significant increase in resting vagally mediated heart rate variability following exercise, which mediated treatment effects, suggesting an enhanced ability to cope with stress (Zeibig et al., 2023). Research on the effects of exercise on repetitive negative thinking in transdiagnostic samples is also limited, with only one study demonstrating that single bouts of moderate exercise reduce repetitive negative thinking in inpatients with various mental disorders, including depression, substance use disorder, bipolar disorder, and anxiety disorders (Brand et al., 2018). Furthermore, we are only aware of one study examining the effects of exercise on sleep quality in a transdiagnostic sample, which showed significant improvements compared to a passive control group (Zeibig et al., 2021).

In addition to the currently limited evidence from transdiagnostic samples, severe methodological shortcomings in studies with both disorder-specific and transdiagnostic samples include the reliance on small sample sizes and the sparse use of mediation models, particularly those that simultaneously incorporate multiple transdiagnostic mechanisms. Since many transdiagnostic mechanisms are interrelated and share variance with each other, it is crucial to examine them jointly within a single mediation analysis. This approach allows for estimating the unique contribution of each mechanism while controlling for their overlap. Therefore, we aimed to address this research gap by investigating how the mechanisms – perceived stress, repetitive negative thinking, and sleep quality – jointly mediate the effect of exercise on global transdiagnostic symptom severity, assessing them together in a single model within a large trial. Notably, in a cross-sectional analysis using baseline data from the current transdiagnostic sample, both higher levels of repetitive negative thinking and worse sleep quality were significantly and independently associated with greater global symptom severity, whereas stress perception was not analyzed (Frei et al., 2025). The present study reports the 6- and 12-month follow-up data as a secondary analysis of the ImPuls trial (Wolf et al., 2021; Wolf et al., 2024). ImPuls is a transdiagnostic group exercise intervention for mental health outpatients with depressive disorders, agoraphobia, panic disorder, PTSD, and/or nonorganic primary insomnia. The following hypotheses were tested: (1) the ImPuls plus treatment-as-usual condition will show a greater reduction in repetitive negative thinking and perceived stress, and a greater increase in sleep quality compared to treatment-as-usual alone, at 6 and 12 months and (2) the treatment effect of ImPuls in addition to treatment-as-usual on the reduction of global symptom severity will be partially mediated by a reduction in repetitive negative thinking and perceived stress, and by an increase in sleep quality, at 6 and 12 months.

## Methods

### Study design

This report is a secondary analysis of the ImPuls trial (Wolf et al., 2021; Wolf et al., 2024), a pragmatic, multisite, block-randomized controlled trial with two parallel groups (i.e. ImPuls plus treatment-as-usual vs. treatment-as-usual) that included assessments at baseline as well as 6 and 12 months after randomization. The ImPuls trial was conducted according to the guidelines of the Declaration of Helsinki of 2010 and was approved by the local ethics committee for medical research at the University of Tübingen (ID: 888/2020B01, 02/11/2020). The study was registered in the German Clinical Trial Register (ID: DRKS00024152, 05/02/2021). The selection of mental disorders and all transdiagnostic mediators was prespecified in the preregistered general study protocol of the ImPuls trial (Wolf et al., 2021; Wolf et al., 2024). Although the current secondary analyses were preregistered several months after the initial trial registration, this occurred prior to conducting any data analysis. The statistical analysis plan was preregistered on Open Science Framework (https://osf.io/p7kxn) before data access. The dataset used for the current study was released by an external data manager only after the preregistration had been completed. Whereas the study results regarding the transdiagnostic efficacy were already available, these did not include any analyses of the mediators examined in the current report; consequently, no prior knowledge of the effects of the mediator outcomes was available.

### Participants

Participants were recruited from 10 different institutions and included if the following criteria were fulfilled: age between 18 and 65 years, fluent in German, insured by one of the two cooperating health insurance providers, Allgemeine Ortskrankenkasse Baden-Württemberg or Techniker Krankenkasse, no medical contraindications for exercise (participants needed to confirm their ability to exercise through a medical consultation prior to the intervention) and at least one of the following diagnoses according to the International Classification of Diseases (10th ed.) (World Health Organization, 2004): major depressive disorders (F32.1, F32.2, F33.1, F33.2), agoraphobia (F40.0, F40.01), panic disorder (F41.0), PTSD (F43.1), and insomnia (F51.0). Exclusion criteria were acute mental and behavioral disorders due to psychotropic substances (F10.0–F19.9, except harmful use, for example, F10.1, which was allowed), acute eating disorders (F50), acute bipolar disorder (F31), acute schizophrenia (F20), acute suicidality, regular engagement in at least 30 minutes of exercise of at least moderate intensity more than once a week, continuously over a 6-week period within the 3 months prior to the structured clinical interview used to confirm the study diagnosis, and any medical contraindication to exercise determined by a general practitioner or a specialized medical professional.

Given the pragmatic, real-world design of the trial, the selection of included mental disorders focused on those for which exercise had already shown therapeutic efficacy. At the time of the main trial’s registration, evidence for the effectiveness of exercise in treating other disorders – such as obsessive-compulsive disorder or somatoform disorders (Freedman & Richter, 2021; Kroenke, 2007) – was still limited. These conditions were therefore not prioritized as target diagnoses but were not explicitly excluded. The detailed comorbid diagnoses included in the study are presented in Supplementary File S1.

A total of 400 participants were randomly assigned to either the ImPuls plus treatment-as-usual group (*n* = 199) or the treatment-as-usual alone group (*n* = 201) (see the Supplementary File S2 and Wolf et al. (2024) for a detailed flow of participants). Baseline demographic and clinical characteristics of the sample are shown in Table 1. The mean age was 42.20 years (*SD* = 13.23, range = 19–65) and 71.2% identified as female. Based on the Structured Clinical Interview for DSM-5 Disorders (American Psychiatric Association, 2022; Beesdo-Baum, Zaudig, & Wittchen, 2019), 71.9% of participants were diagnosed with depression (*n* = 287), 11.5% with panic disorder (*n* = 46), 9.3% with agoraphobia (*n* = 37), 18.0% with PTSD (*n* = 72), and 20.3% with primary insomnia (*n* = 81). Ninety-eight participants (24.6%) additionally had at least one of the other inclusion diagnoses.

**Table 1.** Baseline demographic and clinical characteristics of the intention-to-treat sample (*N* = 399)

	ImPuls plus TAU (*n* = 198)	TAU (*n* = 201)	ImPuls plus TAU compared with TAU (*N* = 399)		
	*n (%)*	*n (%)*	*X^2^*	*df*	*p*
Gender					.058^a^
Male	56 (28.3)	50 (24.9)			
Female	141 (71.2)	143 (71.1)			
Diverse	1 (0.5)	8 (4.0)			
Primary diagnosis					
Depression (F32.1, F32.2, F33.1, F33.2)	146 (73.7)	141 (70.2)	0.47	1	.493
Agoraphobia (F40.00, F40.01)	19 (9.6)	18 (9.0)	0.00	1	.962
Panic disorder (F41.0)	25 (12.6)	21 (10.5)	0.28	1	.600
Posttraumatic stress disorder (F43.1)	31 (15.7)	41 (20.4)	1.21	1	.271
Primary insomnia (F51.0)	44 (22.2)	37 (18.4)	0.68	1	.411
Comorbidity					
Any other inclusion diagnosis	53 (26.8)	45 (22.4)	0.81	1	.368
Any other noninclusion psychiatric diagnosis	92 (46.7)	105 (53.3)	0.86	1	.354
Any other inclusion diagnosis or any further psychiatric diagnosis	116 (58.3)	126 (62.7)	0.63	1	.426
Current treatment					
Outpatient psychological treatment^b^	104 (52.5)	109 (54.2)	0.81	1	.370
Outpatient pharmacological treatment^c^	108 (54.6)	108 (53.7)	0.00	1	.949
Outpatient psychological or pharmacological treatment^d^	150 (75.8)	150 (74.6)	0.00	1	1
	*M (SD)*	*M (SD)*	*t*	*df*	*p*
Age (years)^e^	41.73 (12.8)	42.65 (13.6)	0.69	393	.490
Primary outcome					
Global severity index (BSI–18)^f^	22.08 (11.8)	22.01 (10.5)	−0.05	396	.956
Secondary outcomes/mediators					
Perceived stress (PSS)^c^	34.76 (5.6)	35.05 (5.9)	0.50	395	.614
Repetitive negative thinking (PTQ)^e^	38.58 (11.5)	39.68 (10.5)	0.99	393	.321
Sleep quality (PSQI)^g^	10.34 (3.7)	10.06 (3.8)	−0.73	365	.465

*Note:* BSI-18, brief symptom inventory-18 (Franke, 2017); PSS, perceived stress scale (Cohen et al., 1983; Klein et al., 2016); PTQ, perseverative thinking questionnaire (Ehring et al., 2011); PSQI, Pittsburgh sleep quality index (Buysse et al., 1989); TAU, treatment-as-usual.

a Fisher’s exact test was used as a replacement for the chi-square test because the frequency of one cell was less than 5.

b Eighty-six missing values (a high number of missing responses due to an error in the questionnaire regarding current psychological treatment).

c Two missing values.

d Fifty-eight missing values.

e Four missing values.

f One missing value.

g Thirty-two missing values.

### Procedure

Baseline data were collected at 10 study sites in Baden-Württemberg, Germany, from March 2021 to May 2022. Participants were initially contacted by phone to receive project information and complete screenings regarding eligibility and somatic contraindication. Those potentially eligible were invited to the nearest study site for an in-house meeting. They were informed about the study, provided written consent, and were screened initially for exclusion diagnoses to prepare for the following Structured Clinical Interview (Beesdo-Baum et al., 2019) with a trained psychologist. Once six participants per study site were deemed eligible, they received online questionnaires via the web-based data management system REDCap (Harris et al., 2009; Harris et al., 2019), which could be answered within a 14-days period. After having completed these assessments, participants were randomly assigned to the ImPuls plus treatment-as-usual or the treatment-as-usual alone group (Wolf et al., 2024).

### Intervention

ImPuls is a 6-month transdiagnostic group exercise intervention that included supervised group meetings and 30 minutes of moderate-to-vigorous aerobic exercise performed as outdoor running two to three times per week. In the group meetings, behavioral change techniques (e.g. goal setting, barrier management) were delivered by specifically qualified exercise therapists to increase motivation and commitment to maintaining an exercise routine. After a 4-week supervised period, participants continued with nonsupervised aerobic exercise for 5 months. Telephone calls with exercise therapists were used for long-term exercise monitoring over a 5-month period. Treatment-as-usual included any standard intervention typically provided in the German outpatient setting, such as psychological (cognitive behavioral therapy, psychoanalysis, systemic therapy) and pharmacological treatments (Wolf et al., 2021; Wolf et al., 2024).

### Outcomes

#### Primary outcome

Global symptom severity at 6 months assessed with the Global Severity Index (GSI) derived from the German version of the Brief Symptom Inventory-18 (Franke, 2017), served as the primary outcome and endpoint of the efficacy study (Wolf et al., 2021; Wolf et al., 2024). Therefore, the GSI at both 6 months post intervention and 12 months follow-up were utilized as primary outcome in the mediation analyses conducted in the present study. The GSI encompasses ratings of general mental distress across somatization, depression, and anxiety symptom scales. Each symptom scale consists of six items, resulting in a total of 18 items that were rated on a 5-point Likert scale (range: 0–4). The total score for each scale is calculated, and the GSI is obtained by summing these three scores. Higher scores on the GSI indicate greater levels of distress, with a clinical cut-off set at 12. In German outpatients with various mental disorders, the GSI has shown good internal consistency (Cronbach’s α = .89) and construct validity in patients with affective disorders and anxiety disorders (Spitzer et al., 2011). The primary outcome was assessed in the intention-to-treat sample.

#### Mediators

Perceived stress was assessed with the German version of the Perceived Stress Scale (S. Cohen, Kamarck, & Mermelstein, 1983; Klein et al., 2016). Ten items measure the degree to which life in the past month has been experienced as unpredictable, uncontrollable, and overwhelming on a 5-point Likert scale (range: 0–4). Higher scores indicate greater perceived stress.

Repetitive negative thinking was assessed with the German version of the Perseverative Thinking Questionnaire (PTQ) (Ehring et al., 2011). The PTQ consists of 15 items evaluating process characteristics of repetitive negative thinking with three items each (repetitive, intrusive, difficult to disengage from, unproductive, capturing mental capacity). The items are rated on a 5-point Likert scale (range: 0–4) and higher scores indicate higher levels of repetitive negative thinking.

Sleep quality was assessed with the global sleep quality score of the German version of the Pittsburgh Sleep Quality Index (Buysse et al., 1989). The global sleep quality score is the sum of seven sleep component scores (range: 0–3), including subjective sleep quality, sleep latency, sleep duration, habitual sleep efficiency, sleep disturbances, use of sleeping medications, and daytime dysfunction. Higher scores indicate worse sleep quality. Indices of validity and reliability for the included measurement instruments are provided in Supplementary File S3. All secondary outcomes and mediators were assessed at 6 and 12 months in the intention-to-treat sample.

##### Choice of mediators

The mediators were selected a priori based on theoretical considerations from established transdiagnostic frameworks, representing core processes targeted by the intervention. Furthermore, they are considered exercise-responsive and intervention-relevant, reflecting processes explicitly targeted by the intervention (i.e. reduction of stress and rumination, or improvement in sleep), rather than broader psychological constructs such as self-efficacy. The mediators also exhibit temporal sensitivity, being expected to change over the 6-month intervention period, unlike more stable traits such as perfectionism or intolerance of uncertainty. Consistent with the principle of parsimony, additional highly correlated constructs (e.g. emotional intelligence or emotion regulation) were not included, reducing model complexity and maintaining interpretability (Hayes, 2022).

### Statistical analysis

Data preparation and analysis were carried out using R, version 4.4.1 (R Core Team, 2024). The analytic code and data are available at https://osf.io/5rcuz/files/osfstorage. Descriptive statistics were used to analyze sample characteristics and are reported in frequencies (*n*) and percentages (%) for categorical variables and in means (*M*) and standard deviations (*SD*) for continuous variables.

Analyses comparing the changes in the mediators between groups (first hypothesis) were conducted on the intention-to-treat sample using the full-information maximum likelihood estimation available in the R package *lme4* for linear mixed models (LMMs) (Bates, Mächler, Bolker, & Walker, 2015). Models included study condition (i.e. ImPuls plus treatment-as-usual vs. treatment-as-usual), assessment points (i.e. baseline, 6 months, 12 months) and their interaction as fixed effects and a random intercept for subjects. Random slopes were set to zero in the estimation process due to singular fits and consequently not included in the analysis. Repetitive negative thinking, sleep quality, and perceived stress served as outcomes and were analyzed through three separate LMMs. Assumptions of LMMs (i.e. linearity, normality of the residuals, homoscedasticity, and multicollinearity) were visually inspected or verified by a statistical test (i.e. Variance Inflation Factor for multicollinearity with a threshold of >10 indicating multicollinearity). Post hoc tests for differences between study conditions at 6 and 12 months after randomization were conducted using two-tailed *t*-tests for independent samples. Effect sizes were determined using Cohen’s *d* (J. Cohen, 1988) based on estimated marginal means, and statistical significance was defined as a *p* value *<.*05.

Mediation analyses (second hypothesis) were performed on the intention-to-treat sample using the full-information maximum likelihood estimation provided in the R package *lavaan* for structural equation modeling (SEM) (Rosseel, 2012). To evaluate the mediating role of changes in perceived stress, repetitive negative thinking, and sleep quality on treatment effects, we used a path model specified in the framework of SEM. Study condition as either ImPuls plus treatment-as-usual versus treatment-as-usual served as the predictor, simple change scores of perceived stress, repetitive negative thinking, and sleep quality as mediators, and of global symptom severity as the outcome. Two path models were estimated: one for baseline to 6-month changes and one for baseline to 12-month changes. We further computed bootstrapped standard errors for the three (direct, indirect, and total) effects with 5000 iterations. The same mediation analyses were repeated on the completer sample as sensitivity analyses. Completers were operationalized as those who attended at least two complete weeks within the 4-week supervised intervention period. Again, statistical significance was defined as a *p* value *<.*05.

## Results

### Primary and secondary outcomes

At baseline, 39 (2.4%) values of primary and secondary outcomes were incomplete, at 6-month 150 (9.4%), and at 12-month assessment 156 (9.8%).

The interaction effect of study condition by assessment point was statistically significant for perceived stress, *F*(2, 687.05) = 7.30, *p* = .001, repetitive negative thinking, *F*(2, 680.31) = 24.53, *p* < .001, and sleep quality, *F*(2, 644.66) = 4.74, *p* = .009. Post hoc analyses of differences between study conditions at both the 6- and 12-month assessment revealed lower repetitive negative thinking and perceived stress in the intervention group compared to the control group at both assessment points. Effects were statistically significant, with small to large effects sizes (first hypothesis). No effects were found for sleep quality. Table 2 shows the results of all LMMs.

**Table 2.** Marginal means, standard deviations, confidence intervals, effect sizes, and results of linear mixed-model analyses

Measure and assessment point	ImPuls plus TAU (*n* = 198)			TAU (*n* = 201)			*d* ^a^	*p*	*B* ^b^	Change from baseline in ImPuls plus TAU compared with TAU		
	Mean	SD	95% CI	Mean	SD	95% CI				95% CI	*d* ^b^	*p*
Perceived stress (PSS)												
Baseline	34.76	6.59	33.84, 35.68	35.05	6.55	34.14, 35.96						
6 months	30.73	6.81	29.69, 31.76	33.06	6.67	32.14, 33.99	−0.35	.001	−2.05	−3.37, −0.72	−0.47	.002
12 months	29.54	6.87	28.48, 30.60	32.19	6.60	31.27, 33.11	−0.39	<.001	−2.36	−3.70, −1.02	−0.54	.001
Repetitive negative thinking (PTQ)												
Baseline	38.58	12.58	36.82, 40.34	39.68	12.52	37.95, 41.41						
6 months	27.82	12.82	25.87, 29.76	35.95	12.65	34.19, 37.72	−0.64	<.001	−7.04	−9.33, −4.75	−0.93	<.001
12 months	27.43	12.88	25.45, 29.42	35.58	12.57	33.83, 37.34	−0.64	<.001	−7.05	−9.37, −4.73	−0.93	<.001
Sleep quality (PSQI)												
Baseline	10.26	3.91	9.72, 10.81	9.92	3.86	9.39, 10.46						
6 months	8.62	3.92	8.03, 9.22	9.30	3.89	8.76, 9.84	−0.17	.106	−1.01	−1.74, −0.28	−0.43	.007
12 months	8.40	3.96	7.79, 9.00	9.03	3.87	8.50, 9.57	−0.16	.132	−0.98	−1.72, −0.23	−0.42	.010

*Note:* PSS, perceived stress scale, PTQ, perseverative thinking questionnaire, PSQI, Pittsburgh sleep quality index, TAU, treatment-as-usual.

a Cohen’s *d* for 6- and 12-month treatment effect.

b *B* = assessment point × study condition, *d* = Cohen’s *d* for the interaction effect.

**Figure 1.**
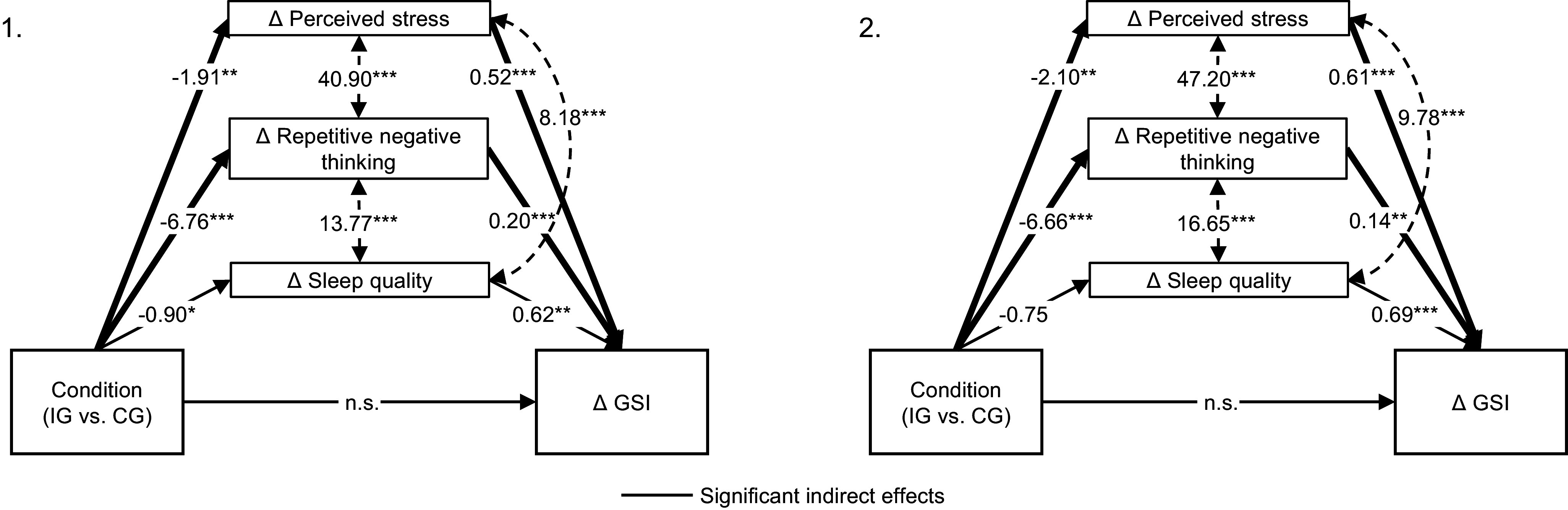
Results of mediation analysis for baseline to 6-month changes (1) and to 12-month changes (2). GSI = Global Severity Index measured with the Brief Symptom Inventory-18. IG (intervention group) = ImPuls plus treatment-as-usual; CG (control group) = treatment-as-usual alone. Path coefficients are unstandardized. ***p < .001, **p < .01, *p < .05.

Results of the mediation analyses (second hypothesis) indicated significant indirect effects on changes in global symptom severity from baseline to 6- and 12-month assessments, mediated by changes in perceived stress (baseline to 6 months: β = −0.99, *p* = .024; baseline to 12 months: β = −1.28, *p* = .014) and repetitive negative thinking (baseline to 6 months: β = −1.34, *p* = .004; baseline to 12 months: β = −0.94, *p* = .024). Changes in sleep quality were no significant mediator. The direct effect of the condition was no longer significant in both path models from baseline to 6- and 12-month assessments, suggesting full mediations. Due to saturated models, no fit indices are applicable. The detailed results of the mediation analyses are shown in Figure 1 and Supplementary File Table S4.

To evaluate potential overlap between the mediators (i.e. perceived stress, repetitive negative thinking, and sleep quality) and the outcome, we examined bivariate correlations at corresponding assessment points (i.e. baseline, 6- and 12-month follow-up), as well as correlations between their respective change scores. The analyses revealed consistently high yet acceptable levels of association for all mediators and change scores. Importantly, a significant association between mediator and outcome is generally considered a necessary condition for mediation, as it reflects the indirect path in the mediation model (Hayes, 2022). The detailed results are shown in Supplementary File S5.

### Sensitivity analysis

Sensitivity analyses with completers only in the ImPuls plus treatment-as-usual condition (*n* = 161) revealed similar results for both the first and second hypotheses, including the LMM and mediation analyses. Detailed results of the sensitivity analysis are provided in Supplementary File S6.

## Discussion

The aim of the present study was to examine perceived stress, repetitive negative thinking, and sleep quality as underlying mechanisms of the treatment effects of an exercise intervention for mental health outpatients with depressive disorders, agoraphobia, panic disorder, PTSD, and/or nonorganic primary insomnia. As expected, the exercise intervention plus treatment-as-usual was efficacious in reducing perceived stress and repetitive negative thinking from baseline to 6- and 12-month assessment when compared to treatment-as-usual alone. Between-group differences at 6 and 12 months were significant, with small to medium effects for perceived stress and medium to large effects for repetitive negative thinking, even at 12-month follow-up. Reductions in perceived stress and repetitive negative thinking from baseline to both follow-up assessments fully mediated the intervention effect on global symptom severity. We did not find a mediation effect of sleep quality.

Perceived stress and repetitive negative thinking are clinically closely related, as evidenced by the high estimates of the direct effects between the changes of the mediators in the path models specified in the framework of SEM. Our study highlights the interrelatedness of these transdiagnostic processes but reveals that changes in perceived stress and repetitive negative thinking independently mediate the intervention effect on global symptom severity.

Interestingly, exercise has been shown to increase resting vagally mediated heart rate variability in patients with mental disorders, reflecting enhanced capacity for stress coping or potentially reduced stress reactivity (Zeibig, Takano, et al., 2023). These findings, along with the results of the present study, can be interpreted through the lens of the cross-stressor adaptation hypothesis (Sothmann, 2006; Sothmann et al., 1996). This hypothesis posits that exposure to one type of stressor may improve an individual’s ability to cope with a range of other stressors by directly promoting adaptive changes in stress reactivity. Given that exercise elicits physiological responses similar to those of external stressors – such as increased heart rate and release of cortisol and adrenaline – it suggests that regular physical activity may promote beneficial psychophysiological adaptations in the human stress response system. These adaptations could reduce stress reactivity (Mücke, Ludyga, Colledge, & Gerber, 2018) and ultimately improve the transdiagnostic symptomatology of mental disorders as a basic, bottom-up physiological process that is more independent of cognitive processes.

Repetitive negative thinking is considered not only a dysfunctional strategy for coping with stress, but also as a general cognitive pattern – encompassing negative emotions, maladaptive memories, and worry – which may explain its independent mediation effect on global symptom severity. Individuals suffering from mental health conditions tend to focus on and repeatedly think about their moods, emotions, symptoms, or negative life events as well as their underlying causes (Whitmer & Gotlib, 2013). This narrowed focus limits access to alternative, more adaptive thoughts, which restricts the use of effective strategies and thereby perpetuating unpleasant feelings, thoughts, and symptoms. One way to break this vicious cycle is to distract oneself from repetitive negative thoughts. The ‘Distraction Hypothesis’ (Kent, 2006; Nolen-Hoeksema, 1991) suggests that exercise may have this potential by deliberately shifting attention away from symptoms and their potential causes or consequences, toward engaging in a pleasurable or energy-consuming activity. Recent studies testing the distraction hypothesis show that aerobic exercise with moderate intensity both as a single bout (Welkerling et al., 2026) and as a 9-week intervention with three sessions per week (Craft, 2005) reduces repetitive negative thinking in patients with depression. Thus, patients in the current study may have used exercise as a strategy to distract themselves from negative thoughts and feelings, facilitating the (re)accessing of more adaptive thoughts or coping strategies and, in turn, improving symptomatology. Similarly, a recent study found that the intentional use of exercise for affect regulation mediates the treatment effects of exercise interventions on mental health in psychiatric outpatients (Zeibig et al., 2023).

ImPuls did not improve sleep quality, which is in line with recent evidence suggesting that whereas exercise has robust effects on depression, its impact on PTSD and anxiety disorders is less conclusive (Szuhany, Sullivan, Gills, & Kredlow, 2024). Consequently, sleep quality may be a particularly important mechanism of change in depression and insomnia, but not in a transdiagnostic sample that also includes anxiety disorders and PTSD.

In the current study, perceived stress and repetitive negative thinking fully mediated the treatment effect of ImPuls on global symptom severity. However, other interrelated mediators may also be relevant. Previous studies suggest that increases in self-reported exercise volume (Wolf et al., 2024; Zeibig et al., 2021), and increased exercise-related self-efficacy as well as self-esteem (Nguyen Ho et al., 2023; Rodriguez-Ayllon et al., 2023) mediate the treatment effects of exercise interventions on mental health in psychiatric outpatients and healthy individuals. In addition to identifying more potential mediators, future research should examine the temporal dynamics and complex causal interactions of these factors. For example, exercise may initially and immediately serve as a deliberate distraction from repetitive negative thinking (Welkerling et al., 2026) and enhance positive affect (Bourke et al., 2022), which could reinforce future engagement in regular exercise (Rhodes & Kates, 2015). Continued engagement in exercise, along with beneficial affective changes, may promote new coping strategies for stress, enhance exercise-related or general self-efficacy, reduce stress perception, and ultimately improve symptomatology. These dynamics could be explored through detailed, fine-grained longitudinal studies. Ecological momentary assessment, which captures a wide range of psychophysiological parameters, would be particularly valuable for investigating how exercise affects stress reactivity, stress perception, and repetitive negative thinking – or other potential mediators – in the context of daily challenges, as well as their causal dynamics.

Importantly, the extent to which the selected disorders can be considered transdiagnostic is limited. Depression, anxiety disorders, PTSD, and insomnia exhibit substantial symptom overlap, including shared features such as sleep disturbances, hyperarousal, and negative affect. However, this overlap does not contradict a transdiagnostic framework; rather, it supports it by highlighting shared underlying mechanisms such as perceived stress, repetitive negative thinking, and sleep quality (Dalgleish et al., 2020; Sauer-Zavala et al., 2017). Moreover, transdiagnostic research commonly includes disorders with overlapping features that remain distinct in diagnostic systems (e.g. DSM-5, ICD-10), reflecting current clinical and diagnostic practice (Fusar-Poli et al., 2019). Finally, although these disorders share common features, their core symptom profiles remain distinct – for example, persistent low mood in depression, intrusive memories in PTSD, or panic attacks in panic disorder – underscoring the importance of addressing both shared and disorder-specific aspects within a transdiagnostic framework (Cuijpers et al., 2023; Dalgleish et al., 2020).

Our study has several strengths and limitations. The large sample size is a significant strength, as it enabled the conduct of complex mediation analyses involving three potential mechanisms of change. The simultaneous assessment of these three transdiagnostic mechanisms underlying the treatment effects of exercise on global symptom severity, evaluated as mediators within a single model, should also be emphasized as a key advantage, given that this approach remains rare in the existing literature. Many studies to date have concentrated on physiological mechanisms or, when examining psychological processes, have excluded participants with mental health conditions or investigated only a single mechanism (Bendau et al., 2024; Precht, Margraf, Stirnberg, & Brailovskaia, 2023). Therefore, our study adds to the expanding body of research by concentrating on prevalent, interrelated psychological processes. Furthermore, the inclusion of a transdiagnostic sample of participants with various highly comorbid mental disorders, along with the use of a transdiagnostic outcome, is a notable strength of this study and aligns with the growing evidence supporting transdiagnostic approaches to mental health problems (Dalgleish et al., 2020). Additionally, the high level of standardization in the trial (Wolf et al., 2021; Wolf et al., 2024) enhances the reliability and validity of the transdiagnostic mediators by ensuring consistent measurement and reducing variability, which is crucial for drawing reliable and valid conclusions. Finally, the hypotheses investigated in the current study were preregistered prior to database lock. The study also faces several limitations that must be considered when interpreting the results. First, although we conducted mediation analyses, definitive conclusions about causal relationships cannot be drawn as mediators and outcomes were assessed simultaneously at each assessment point (Gelfand, Mensinger, & Tenhave, 2009). Second, most participants (72%) were diagnosed with depressive disorders, whereas conditions such as agoraphobia and panic disorder were less prevalent. Although this distribution reflects the reality of outpatient mental healthcare in Germany, the magnitude or relevance of transdiagnostic mechanisms may depend on the specific composition of the sample. For instance, other mechanisms, such as anxiety sensitivity (Jacquart et al., 2019), may play a more prominent role in samples primarily composed of individuals with anxiety disorders. Third, we noted differing dropout rates across groups, with higher dropout observed in the ImPuls plus treatment-as-usual condition. Comparing these rates directly is challenging due to the financial compensation provided exclusively to participants in the control condition, which may have contributed to its lower attrition rate. To address missing data concerns, we used LMMs and SEM, which are recognized as valid and unbiased approaches for handling data assumed to be missing at random (Bell, Kenward, Fairclough, & Horton, 2013; Rosseel, 2012). Fourth, as insomnia symptoms are diagnostic criteria for depression, PTSD, and insomnia, and sleep quality was investigated as a potential mechanism of change, it is not possible to fully disentangle symptom reduction from underlying process change. Fifth, it should be noted that the transdiagnostic primary outcome (GSI) is composed of three subscales that reflect disorder-specific symptom domains (i.e. somatization, depression, anxiety). Consequently, individuals scoring highly on one of these subscales are likely to exhibit elevated scores on the overall GSI. This structure may limit the measure’s ability to fully capture transdiagnostic symptomatology. Therefore, future research might consider or develop alternative outcome measures that more comprehensively reflect transdiagnostic constructs. Sixth, the sample was restricted to physically inactive individuals, as those already meeting the targeted level of physical exercise intended by the ImPuls intervention (i.e. two to three sessions per week of 30 minutes of outdoor exercise) were excluded. This limits the generalizability of our findings. Finally, since we compared ImPuls plus treatment-as-usual versus treatment-as-usual alone, the study lacked an active control group. As a result, we cannot rule out the potential influence of nonspecific factors – such as increased attention from study staff or participants’ expectations – on both the outcomes and mechanisms of change. Given the real-world, pragmatic design of the trial, it is not possible to determine whether ImPuls exerts similar, superior, or distinct effects on transdiagnostic mechanisms compared to other interventions, such as cognitive behavioral therapy. Moreover, it remains unclear whether the observed effects are due to ImPuls as an add-on to standard interventions or as an add-on to no treatment at all – and whether this distinction impacts outcomes. Consequently, whereas treatment-as-usual control groups enhance ecological validity, they limit the capacity to isolate the specific mechanisms of action associated with the intervention.

## Conclusion

Our research indicates that changes in perceived stress and repetitive negative thinking act as transdiagnostic mediators by fully mediating the treatment effect of an exercise intervention on global symptom severity in mental health outpatients with depressive disorders, agoraphobia, panic disorder, PTSD, and/or nonorganic primary insomnia. Future studies should investigate the temporal dynamics and causal relationships between potential mediators underlying the treatment effects of exercise interventions on mental health.

## Supporting information

10.1017/S0033291725103085.sm001 Frei et al. supplementary material Frei et al. supplementary material

## Data Availability

The data that support the findings of this study are openly available in Open Science Framework at https://osf.io/5rcuz/files/osfstorage.

## Supplementary material

The supplementary material for this article can be found at http://doi.org/10.1017/S0033291725103085.
