# Supplementary material for: Changes in repetitive negative thinking and stress perception mediate treatment effects of a transdiagnostic exercise intervention
Source: Psychol Med. 2026 Jan 9;56:e10. doi: 10.1017/S0033291725103085 (PMC12885332; doi:10.1017/S0033291725103085)
Supplement: Frei et al. supplementary material [file S0033291725103085sup001.zip › S1_Comorbidities.docx]

**S1.** Comorbid diagnoses (*N* = 399)

|  | *n* (%) |
| --- | --- |
| Mental and behavioral disorders due to harmful use of alcohol (F10.1, F10.10) | 11 (2.8) |
| Mental and behavioral disorders due to harmful use of cannabinoids (F12.1, F12.10) | 5 (1.3) |
| Mild depressive disorder (F32.0, F32.4) | 11 (2.8) |
| Premenstrual dysphoric disorder (F32.81) | 9 (2.3) |
| Mild or partially remitted recurrent depressive disorder (F33.0, F33.4) | 21 (5.3) |
| Dysthymia (F34.1) | 83 (20.8) |
| Social phobia anxiety disorders (F40.1, F40.10) | 34 (8.5) |
| Specific (isolated) phobias (F40.2) | 29 (7.3) |
| Generalized anxiety disorder (41.1) | 14 (3.5) |
| Obsessive-compulsive disorders (F42.0, F42.1, F42.2, F42.3, F42.4) | 23 (5.8) |
| Somatoform disorders (F45.0, F45.1, F45.2, F45.41) | 15 (3.8) |
| Hyperkinetic disorders (F90.0, F90.1, F90.2) | 11 (2.8) |
