# Supplementary material for: Changes in repetitive negative thinking and stress perception mediate treatment effects of a transdiagnostic exercise intervention
Source: Psychol Med. 2026 Jan 9;56:e10. doi: 10.1017/S0033291725103085 (PMC12885332; doi:10.1017/S0033291725103085)
Supplement: Frei et al. supplementary material [file S0033291725103085sup001.zip › S2_Patient Flow.docx]

Telephone screen for eligibility (n = 1284)

Excluded (n = 609)

♦ Meeting exclusion criteria (n = 226)

- Outside of age range (n = 18)
- Non-participating health insurer (n = 117)
- Too much physical activity (n = 91)

♦ Other reasons^1^ (n = 383)

Randomization (n = 400)

Excluded (n = 199)

♦ Not meeting criteria for any diagnosis needed for inclusion (n = 124)

♦ Meeting criteria for at least one diagnosis leading to exclusion (n = 34)

♦ No further interest (n = 10)

♦ Physical contraindication (n = 3)

♦ Other reasons^1^ (n = 28 )

Baseline (n = 401)

Withdrawn during pre assessment

♦ Declined to participate (n = 1)

Diagnostic interview (n = 600)

Initial interview (n = 675)

Excluded (n = 75)

♦ Not meeting inclusion criteria (n = 7)

♦ Physical contraindication (n = 2)

♦ Meeting criteria for at least one diagnosis leading to exclusion (n = 6)

♦ No further interest in participation (n = 16)

♦ Other reasons^1^ (n = 44)

## Enrollment

**Treatment-as-usual** (n = 201)

**ImPuls plus treatment-as-usual** (n = 199)

♦ Received allocated intervention (n = 161)

♦ Did not receive allocated intervention^2^ (n = 38)

6-month assessment

♦ Participated (n = 193)

♦ Study Dropout^2^ (n = 4)

♦ Lost to post (n = 2)

6-month assessment

♦ Participated (n = 155)

♦ Study Dropout^2^ (n = 42)

♦ Lost to post (n = 3)

12-month follow-up assessment

♦ Participated (n = 195)

♦ Study Dropout^2^ (n = 2)

12-month follow-up assessment

♦ Participated (n = 143)

♦ Study Dropout^2^ (n = 14)

Analysed (n = 198)

♦ Excluded from analysis due to withdrawal of informed consent (n = 1)

Analysed (n = 201)

**Fig. S2.** Flow of participants.

^1^Other reasons include organizational problems, relocation, no more contact possible, physical constraints, language problems, unknown.
^2^Reasons for loss of participants/data include treatment dropout due to physical adverse event, no further interest, excessive demand of intervention, other or study dropout due to serious adverse event, physical/psychological adverse event, no further interest, excessive demand of research-specific tasks or intervention, request of data deletion, other/lost to follow-up assessment.
