# Supplementary material for: Changes in repetitive negative thinking and stress perception mediate treatment effects of a transdiagnostic exercise intervention
Source: Psychol Med. 2026 Jan 9;56:e10. doi: 10.1017/S0033291725103085 (PMC12885332; doi:10.1017/S0033291725103085)
Supplement: Frei et al. supplementary material [file S0033291725103085sup001.zip › S3_Indices of validity and reliability.docx]

**S3.** Indices of validity and reliability for the included measurement instruments

The Perceived Stress Scale [PSS] (Cohen, Kamarck, & Mermelstein, 1983; Klein et al., 2016) has demonstrated good internal consistency (Cronbach’s alpha = .84) and construct validity in a representative German community sample (Klein et al., 2016). The Perseverative Thinking Questionnaire [PTQ] has shown an excellent internal consistency (Cronbach’s alpha = .95) among a transdiagnostic clinical sample (Ehring et al., 2011). Additionally, the PTQ has demonstrated significant convergent validity with established measures of repetitive negative thinking as well as substantial predictive validity for symptoms of depression and anxiety disorders in clinical samples (Ehring et al., 2011). The Pittsburgh Sleep Quality Index [PSQI] (Buysse, Reynolds, Monk, Berman, & Kupfer, 1989) has shown acceptable internal consistency (Cronbach’s alpha = .75) and validity in the general German population (Hinz et al., 2017).

References

Buysse, D. J., Reynolds, C. F., 3rd, Monk, T. H., Berman, S. R., & Kupfer, D. J. (1989). The Pittsburgh Sleep Quality Index: a new instrument for psychiatric practice and research. *Psychiatry Research, 28*(2), 193-213. doi:10.1016/0165-1781(89)90047-4

Cohen, S., Kamarck, T., & Mermelstein, R. (1983). A global measure of perceived stress. *Journal of Health and Social Behavior, 24*(4), 385-396. doi:10.2307/2136404

Ehring, T., Zetsche, U., Weidacker, K., Wahl, K., Schonfeld, S., & Ehlers, A. (2011). The Perseverative Thinking Questionnaire (PTQ): validation of a content-independent measure of repetitive negative thinking. *Journal of Behavior Therapy and Experimental Psychiatry, 42*(2), 225-232. doi:10.1016/j.jbtep.2010.12.003

Hinz, A., Glaesmer, H., Brähler, E., Löffler, M., Engel, C., Enzenbach, C., . . . Sander, C. (2017). Sleep quality in the general population: psychometric properties of the Pittsburgh Sleep Quality Index, derived from a German community sample of 9284 people. *Sleep Medicine, 30*, 57-63. doi:10.1016/j.sleep.2016.03.008

Klein, E. M., Brahler, E., Dreier, M., Reinecke, L., Muller, K. W., Schmutzer, G., . . . Beutel, M. E. (2016). The German version of the Perceived Stress Scale - psychometric characteristics in a representative German community sample. *BMC Psychiatry, 16*, 159. doi:10.1186/s12888-016-0875-9
