# Supplementary material for: Changes in repetitive negative thinking and stress perception mediate treatment effects of a transdiagnostic exercise intervention
Source: Psychol Med. 2026 Jan 9;56:e10. doi: 10.1017/S0033291725103085 (PMC12885332; doi:10.1017/S0033291725103085)
Supplement: Frei et al. supplementary material [file S0033291725103085sup001.zip › S4_Hypothesis 2.docx]

**S4.** Results of structural equation modeling with bootstrapping (5000 iterations) on the intention-to-treat sample for model on changes of global symptom severity from baseline to 6- and 12-months assessment. Missings were handled with full-information maximum likelihood estimation.

|  | | | **6-months assessment** | | | |  | | **12-months assessment** | | | | |  |
| --- | --- | --- | --- | --- | --- | --- | --- | --- | --- | --- | --- | --- | --- | --- |
| **Causal Relationships** | |  | *Est* | *SE* | *z* | *p* | |  | | *Est* | *SE* | *z* | *p* | |
| *Direct effects* |  | |  |  |  |  | |  | |  |  |  |  | |
| Δ GSI | ~ Condition (ImPuls plus TAU vs. TAU) | | -1.68 | 1.00 | -1.69 | .090 | |  | | -1.31 | 1.10 | -1.19 | .234 | |
| Δ GSI | ~ Δ Perceived stress | | 0.52 | 0.12 | 4.30 | <.001 | |  | | 0.61 | 0.10 | 6.30 | <.001 | |
| Δ GSI | ~ Δ RNT | | 0.20 | 0.05 | 3.80 | <.001 | |  | | 0.14 | 0.05 | 2.69 | .007 | |
| Δ GSI | ~ Δ Sleep quality | | 0.62 | 0.20 | 3.15 | .002 | |  | | 0.69 | 0.19 | 3.73 | <.001 | |
| Δ Perceived stress | ~ Condition (ImPuls plus TAU vs. TAU) | | -1.91 | 0.71 | -2.69 | .007 | |  | | -2.10 | 0.77 | -2.72 | .007 | |
| Δ RNT | ~ Condition (ImPuls plus TAU vs. TAU) | | -6.76 | 1.30 | -5.25 | <.001 | |  | | -6.66 | 1.42 | -4.68 | <.001 | |
| Δ Sleep quality | ~ Condition (ImPuls plus TAU vs. TAU) | | -0.90 | 0.40 | -2.24 | .025 | |  | | -0.75 | 0.42 | -1.78 | .075 | |
| Δ RNT | ~~ Δ Perceived stress | | 40.90 | 6.01 | 6.80 | <.001 | |  | | 47.20 | 6.22 | 7.59 | <.001 | |
| Δ RNT | ~~ Δ Sleep quality | | 13.77 | 2.64 | 5.21 | <.001 | |  | | 16.65 | 2.76 | 6.03 | <.001 | |
| Δ Perceived stress | ~~ Δ Sleep quality | | 8.18 | 1.57 | 5.23 | <.001 | |  | | 9.78 | 1.66 | 5.88 | <.001 | |
| *Indirect effects* |  | |  |  |  |  | |  | |  |  |  |  | |
| Δ GSI | ~ Condition x Δ Perceived stress | | -0.99 | 0.44 | -2.26 | .024 | |  | | -1.28 | 0.52 | -2.47 | .014 | |
| Δ GSI | ~ Condition x Δ RNT | | -1.34 | 0.47 | -2.88 | .004 | |  | | -0.94 | 0.42 | -2.26 | .024 | |
| Δ GSI | ~ Condition x Δ Sleep quality | | -0.56 | 0.32 | -1.76 | .079 | |  | | -0.52 | 0.34 | 1.54 | .125 | |
| *Total effect* |  | | -4.57 | 1.16 | -3.93 | <.001 | |  | | -163.57 | 90.14 | -1.82 | .070 | |
| *Note.* GSI *=* Global Severity Index, RNT = repetitive negative thinking, TAU = treatment-as-usual. | | | | | | | | | | | | | |  |
