# Supplementary material for: Changes in repetitive negative thinking and stress perception mediate treatment effects of a transdiagnostic exercise intervention
Source: Psychol Med. 2026 Jan 9;56:e10. doi: 10.1017/S0033291725103085 (PMC12885332; doi:10.1017/S0033291725103085)
Supplement: Frei et al. supplementary material [file S0033291725103085sup001.zip › S5_Bivariate correlations.docx]

**S5.** Bivariate correlation matrix of the mediators and outcome as well as their respective change scores from baseline to 6-month and 12-month follow-up

|  | PSS T1 | PSS T2 | PSS T3 | RNT T1 | RNT T2 | RNT T3 | SQ T1 | SQ T2 | SQ T3 |
| --- | --- | --- | --- | --- | --- | --- | --- | --- | --- |
| GSI T1 | 0.57 |  |  | 0.48 |  |  | 0.34 |  |  |
| GSI T2 |  | 0.68 |  |  | 0.62 |  |  | 0.52 |  |
| GSI T3 |  |  | 0.72 |  |  | 0.60 |  |  | 0.53 |
| Change Scores | PSS T1 – T2 | PSS T1 – T3 |  | RNT T1 – T2 | RNT T1 – T3 |  | SQ T1 – T2 | SQ T1 – T3 |  |
| GSI T1 – T2 | 0.52 |  |  | 0.49 |  |  | 0.41 |  |  |
| GSI T1 – T3 |  | 0.56 |  |  | 0.47 |  |  | 0.44 |  |

*Note*. GSI = Global Severity Index assessing global symptom severity, PSS = perceived stress, RNT = repetitive negative thinking, SQ = sleep quality. T1 = baseline, T2 = 6-month follow-up, T3 = 12-month follow-up.
