# Supplementary material for: Changes in repetitive negative thinking and stress perception mediate treatment effects of a transdiagnostic exercise intervention
Source: Psychol Med. 2026 Jan 9;56:e10. doi: 10.1017/S0033291725103085 (PMC12885332; doi:10.1017/S0033291725103085)
Supplement: Frei et al. supplementary material [file S0033291725103085sup001.zip › S6_Sensitivity analysis.docx]

**S6.** Sensitivity analysis on the completer sample

**S.6.1** Marginal means, standard deviations, confidence intervals, effect sizes, and results of linear mixed models analyses

|  | **ImPuls plus TAU (*n* = 161)** | | | **TAU (*n* = 201)** | | |  | |  | |  | | | **Change from baseline in ImPuls plus TAU compared with TAU** | | | | |
| --- | --- | --- | --- | --- | --- | --- | --- | --- | --- | --- | --- | --- | --- | --- | --- | --- | --- | --- |
| **Measure and Assessment Point** | *Mean* | *SD* | *95%CI* | *Mean* | *SD* | *95%CI* |  | *d*^a^ | | *p* | |  | *B*^b^ | | *95%CI* | d^b^ | *p* |  |
| Perceived stress (PSS) |  |  |  |  |  |  |  |  | |  | |  |  | |  |  |  |  |
| Baseline | 34.48 | 6.53 | 33.47, 35.49 | 35.05 | 6.51 | 34.15, 35.95 |  |  | |  | |  |  | |  |  |  |  |
| 6-months | 30.76 | 6.76 | 29.67, 31.85 | 33.06 | 6.62 | 32.14, 33.99 |  | -0.34 | | .002 | |  | -1.74 | | -3.10, -0.37 | -0.40 | .013 |  |
| 12-months | 29.55 | 6.85 | 28.42, 30.67 | 32.19 | 6.56 | 31.28, 33.11 |  | -0.40 | | <.001 | |  | -2.08 | | -3.47, -0.70 | -0.47 | .003 |  |
| Repetitive negative thinking (PTQ) |  |  |  |  |  |  |  |  | |  | |  |  | |  |  |  |  |
| Baseline | 37.49 | 12.49 | 35.56, 39.43 | 39.68 | 12.48 | 37.95, 41.41 |  |  | |  | |  |  | |  |  |  |  |
| 6-months | 27.51 | 12.80 | 25.45, 29.58 | 35.95 | 12.61 | 34.20, 37.71 |  | -0.67 | | <.001 | |  | -6.25 | | -8.57, -3.94 | -0.85 | <.001 |  |
| 12-months | 27.49 | 12.88 | 25.38, 29.60 | 35.58 | 12.52 | 33.83, 37.33 |  | -0.64 | | <.001 | |  | -5.90 | | -8.25, -3.56 | -0.80 | <.001 |  |
| Sleep quality (PSQI) |  |  |  |  |  |  |  |  | |  | |  |  | |  |  |  |  |
| Baseline | 10.14 | 3.91 | 9.53, 10.74 | 9.92 | 3.84 | 9.39, 10.46 |  |  | |  | |  |  | |  |  |  |  |
| 6-months | 8.55 | 3.93 | 7.92, 9.19 | 9.30 | 3.88 | 8.76, 9.84 |  | -0.19 | | .080 | |  | -0.96 | | -1.72, -0.20 | -0.41 | .013 |  |
| 12-months | 8.44 | 3.96 | 7.79, 9.08 | 9.03 | 3.87 | 8.50, 9.57 |  | -0.15 | | .172 | |  | -0.81 | | -1.58, -0.05 | -0.35 | .038 |  |
| *Note.* Completers are defined as those who completed at least 2 full weeks of the supervised intervention period.  PSS = Perceived Stress Scale, PTQ = Perseverative Thinking Questionnaire, PSQI = Pittsburgh Sleep Quality Index.  ^a^Cohen’s *d* for 6- and 12-months treatment effect.  ^b^*B* = time x study condition, *d* = Cohen’s d for the interaction effect | | | | | | | | | | | | | | | | | | |

**S.6.2** Results of structural equation modeling with bootstrapping (5000 iterations) for model on changes of global symptom severity from baseline to 6- and 12-months assessment. Missings were handled with full-information maximum likelihood estimation.

|  | | **6-months assessment** | | | |  | | **12-months assessment** | | | | | | |  |  |
| --- | --- | --- | --- | --- | --- | --- | --- | --- | --- | --- | --- | --- | --- | --- | --- | --- |
| **Causal Relationships** | | *Est* | *SE* | *z* | *p* | |  | | *Est* | *SE* | | *z* | | *p* | | |
| *Direct effects* |  |  |  |  |  | |  | |  |  |  | |  | | |  |
| Δ GSI | ~ Condition (ImPuls plus TAU vs. TAU) | -1.39 | 1.01 | -1.37 | .170 | |  | | -0.97 | 1.14 | -0.85 | | .394 | | |  |
| Δ GSI | ~ Δ Perceived stress | 0.52 | 0.12 | 4.31 | <.001 | |  | | 0.63 | 0.10 | 6.42 | | <.001 | | |  |
| Δ GSI | ~ Δ RNT | 0.19 | 0.05 | 3.57 | <.001 | |  | | 0.14 | 0.06 | 2.41 | | .016 | | |  |
| Δ GSI | ~ Δ Sleep quality | 0.62 | 0.20 | 3.04 | .002 | |  | | 0.63 | 0.19 | 3.38 | | .001 | | |  |
| Δ Perceived stress | ~ Condition (ImPuls plus TAU vs. TAU) | -1.66 | 0.71 | -2.33 | .020 | |  | | -1.92 | 0.79 | -2.43 | | .015 | | |  |
| Δ RNT | ~ Condition (ImPuls plus TAU vs. TAU) | -6.23 | 1.29 | -4.82 | <.001 | |  | | -5.80 | 1.40 | -4.15 | | <.001 | | |  |
| Δ Sleep quality | ~ Condition (ImPuls plus TAU vs. TAU) | -0.82 | 0.43 | -1.93 | .054 | |  | | -0.61 | 0.44 | -1.40 | | .162 | | |  |
| Δ RNT | ~~ Δ Perceived stress | 39.53 | 6.00 | 6.59 | <.001 | |  | | 45.19 | 6.18 | 7.32 | | <.001 | | |  |
| Δ RNT | ~~ Δ Sleep quality | 13.59 | 2.73 | 4.97 | <.001 | |  | | 15.50 | 2.81 | 5.51 | | <.001 | | |  |
| Δ Perceived stress | ~~ Δ Sleep quality | 8.16 | 1.62 | 5.05 | <.001 | |  | | 9.78 | 1.75 | 5.60 | | <.001 | | |  |
| *Indirect effects* |  |  |  |  |  | |  | |  |  |  | |  | | |  |
| Δ GSI | ~ Condition x Δ Perceived stress | -0.86 | 0.44 | -1.98 | .047 | |  | | -1.21 | 0.55 | -2.21 | | .027 | | |  |
| Δ GSI | ~ Condition x Δ RNT | -1.18 | 0.45 | -2.64 | .008 | |  | | -0.79 | 0.39 | -2.03 | | .043 | | |  |
| Δ GSI | ~ Condition x Δ Sleep quality | -0.51 | 0.33 | -1.54 | .124 | |  | | -0.39 | 0.31 | -1.25 | | .211 | | |  |
| *Total effect* |  | -3.95 | 1.21 | -3.28 | .001 | |  | | -133.12 | 93.25 | -1.43 | | .153 | | |  |
| *Note.* GSI *=* Global Severity Index, RNT = repetitive negative thinking, TAU = treatment-as-usual. | | | | | | | | | | | | | | |  |  |
